# Glycogen synthase kinase 3 promotes multicellular development over unicellular encystation in encysting Dictyostelia

**DOI:** 10.1186/s13227-018-0101-6

**Published:** 2018-05-09

**Authors:** Yoshinori Kawabe, Takahiro Morio, Yoshimasa Tanaka, Pauline Schaap

**Affiliations:** 1 School of Life Sciences, University of Dundee, MSI/WTB Complex, Dow Street, Dundee, DD15EH UK; 2 Graduate School of Life and Environmental Sciences, University of Tsukuba, Tsukuba, Ibaraki 305-8572 Japan

**Keywords:** Encystment, Sporulation, Stress response, *Polysphondylium*, Life cycle choice, Glycogen synthase kinase 3, Cell-type specialization, Amoebozoa, Dictyostelia

## Abstract

**Background:**

Glycogen synthase kinase 3 (GSK3) regulates many cell fate decisions in animal development. In multicellular structures of the group 4 dictyostelid *Dictyostelium discoideum*, GSK3 promotes spore over stalk-like differentiation. We investigated whether, similar to other sporulation-inducing genes such as cAMP-dependent protein kinase (PKA), this role of GSK3 is derived from an ancestral role in encystation of unicellular amoebas.

**Results:**

We deleted *GSK3* in *Polysphondylium pallidum*, a group 2 dictyostelid which has retained encystation as an alternative survival strategy. Loss of GSK3 inhibited cytokinesis of cells in suspension, as also occurs in *D. discoideum*, but did not affect spore or stalk differentiation in *P. pallidum*. However, *gsk3*^−^ amoebas entered into encystation under conditions that in wild type favour aggregation and fruiting body formation. The *gsk3*^−^ cells were hypersensitive to osmolytes, which are known to promote encystation, and to cyst-inducing factors that are secreted during starvation. GSK3 was not itself regulated by these factors, but inhibited their effects.

**Conclusions:**

Our data show that GSK3 has a deeply conserved role in controlling cytokinesis, but not spore differentiation in Dictyostelia. Instead, in *P. pallidum*, one of many Dictyostelia that like their solitary ancestors can still encyst to survive starvation, GSK3 promotes multicellular development into fruiting bodies over unicellular encystment.

**Electronic supplementary material:**

The online version of this article (10.1186/s13227-018-0101-6) contains supplementary material, which is available to authorized users.

## Background

Many unicellular protists, including Amoebozoa, survive adverse conditions by shutting down metabolism and differentiating into a walled cyst. Cysts are extremely resilient, which, in case of amoeba pathogens, prevents their eradication by immune clearance or antibiotics [1, 2]. The multicellular Dictyostelia, members of Amoebozoa, evolved an additional strategy to survive starvation stress, in which amoebas aggregate to form a multicellular fruiting structure and differentiate into walled spores and stalk cells. Dictyostelia can be subdivided into four major groups, and while many species in groups 1–3 have retained encystation as an alternative survival strategy, it was lost in group 4, which contains the model organism *Dictyostelium discoideum* [3, 4]. In *D. discoideum*, both secreted and intracellular cyclic AMP (cAMP) play major roles in regulating the multicellular developmental programme. Secreted cAMP, acting on G-protein-coupled cAMP receptors (cARs), acts as a chemoattractant to coordinate aggregation and morphogenesis, and additionally induces prespore differentiation, while inhibiting stalk differentiation. Intracellular cAMP, acting on PKA, triggers the maturation of spore and stalk cells and keeps spore dormant in the fruiting body. cAMP is synthesized by the adenylate cyclases ACA, ACR and ACG, but intracellular levels are critically regulated by the cAMP phosphodiesterase RegA. RegA is activated/inhibited by sensor histidine kinases/phosphatases, which are the targets for signals that control timely spore and stalk maturation and spore germination [5, 6].

Comparative functional analysis of PKA, ACR, ACG and RegA in the group 2 Dictyostelid *Polysphondylium pallidum* and the solitary amoebozoan pathogen *Acanthamoeba castellani* revealed that the intracellular role of cAMP in spore and stalk maturation and spore dormancy is evolutionary derived from a second messenger role in stress-induced encystation [7–11]. PKA, ACR and RegA are deeply conserved in Amoebozoa, and their sequenced genomes contain many sensor histidine kinase/phosphatases, which could act as food/stress sensors, respectively, to regulate RegA [12, 13].

While PKA is required for both the spore and stalk cell differentiation pathways, glycogen synthase kinase 3 (GSK3), a component of the wnt/wingless pathway that regulates many cell fate decisions in metazoa [14, 15], is in *D. discoideum* considered to selectively promote prespore over prestalk differentiation as target for secreted cAMP, which activates GSK3 [16, 17]. We are interested in expanding the range of encystation-inducing proteins that could act as therapeutic targets to prevent encystation of pathogens. We therefore investigated whether, similar to ACR, RegA and PKA, GSK3′s role in sporulation was also evolutionary derived from a role in encystation.

To address this issue we deleted the *GSK3* gene of *P. pallidum*, which, in addition to fruiting body formation, has retained encystation as an alternative survival strategy. Surprisingly, loss of GSK3 had no negative effect on *P. pallidum* sporulation and promoted instead of inhibited encystation.

## Methods

### Growth and development

*Polysphondylium pallidum* (*Pp*), strain PN500, was routinely grown in association with *Escherichia coli or Klebsiella aerogenes* on lactose-peptone (LP) agar. For multicellular development, *Pp* cells were harvested in 20 mM K/K-phosphate, pH 6.5 (KK2), washed free from bacteria and incubated at 10^6^ cells/cm^2^ and 21 °C on non-nutrient agar. To determine growth rate, *Pp* cells were inoculated at 10^5^ cells/ml in KK2 with autoclaved *Klebsiella aerogenes* at OD_600_ = 15.

### Amplification of a *Pp* GSK3 ortholog

The *Pp* *GSK3* gene was amplified by PCR from genomic DNA, using redundant primers GSKredF and GSKredR (Additional file 1: Table 1), which are complementary to amino-acid sequences CHRDIKP and GTPTE/R/KQ, respectively, that are conserved in eukaryote GSK3 proteins. The PCR products were subcloned, and their DNA sequence was determined from 3 independent clones. The complete 1350-bp coding sequence of the *Pp* *GSK3* with 3003-bp 5′ and 1579-bp 3′ UTR was obtained by inverse PCR with primer pair GSKINV1 and GSKINV2 (Additional file 1: Table 1), using religated *Hin*dIII or *Bgl*II-digested *Pp* gDNA as template, respectively. All PCR products were subcloned in pBluescript II KS (-) (Stratagene) or pCR4-XL-TOPO (Invitrogen) and sequenced.

To determine the nucleotide sequence of the *Pp GSK3* mRNAs, polyA^+^ RNA was isolated from *Pp* cells. Full-length cDNAs were subsequently synthesized by RNA-ligation-mediated rapid amplification of 5′ and 3′ cDNA ends (RLM-RACE) and RT-PCR using the GeneRacer kit (Invitrogen) according to the manufacturer’s instructions.

### DNA constructs and transformation

#### Vectors for *GSK3* gene disruption

Partial *GSK3* sequence with 2.2-kb 5′ UTR and 2.9-kb 3′ UTR was amplified by inverse PCR from *Eco*RI-digested and religated *Pp* gDNA, using primers GSKINV3 and GSKINV4 (Additional file 1: Table 1) which contain *Kpn*I sites. The *Kpn*I-digested PCR product was cloned into *Kpn*I-digested pLoxNeoII∆EcoRI, which was generated from pLoxNeoII [10] by destroying its *Eco*RI site by digestion with *Eco*RI, fill-in with Klenow and self-ligation with T4 ligase. This yielded vector pPp-GSK3-KO, which was linearized by *Eco*RI digestion and transformed into *Pp* cells as described previously [18]. The gene disruption was confirmed by Southern blot analysis (Additional file 1: Fig. 1). To remove the Neo cassette, the knockout cells were transformed with pA15NLS.Cre for transient expression of Cre-recombinase [10] and G418-sensitive clones were selected.

#### Complementation of *Pp gsk3*^−^ with *GSK3*

The *GSK3* coding sequence was amplified from cDNA by RT-PCR using primers Pp-GSK3-S51 and Pp-GSK3-E31E (Additional file 1: Table 1) containing *Bgl*II and *Eco*RI sites, respectively. After cloning into pCR4-TOPO (Invitrogen) the PCR product was validated by sequencing, digested with *Bgl*II and *Eco*RI and cloned into *Bgl*II- and *Eco*RI-digested vector pDdNYFP [19], yielding vector pPp-A15GSK3-OE. To express *GSK3* from its own promoter, the promoter region was amplified by PCR using primers Pp-GSK3-51 and Pp-GSK3-31 (Additional file 1: Table 1), cloned into pCR4-TOPO (Invitrogen) and sequenced. After digestion with *Spe*I and *Bgl*II, the 1.5-kb fragment, which contains the *GSK3* promoter region, was cloned into *Nhe*I- and *Bgl*II-digested pPp-A15GSK3-OE. This yielded vector pPp-GSK3-OE, which was introduced into *gsk3*^−^ cells.

### Encystation assay

For quantification of encystation, *Pp* cells were grown in a suspension of autoclaved *K. aerogenes* in KK2, until cell proliferation reached stationary phase. Cells were washed free of bacteria, resuspended in KK2 at 10^7^ cells/ml and shaken at 180 rpm and 21 °C for 48 h. Aliquots of 0.1 ml were sampled at regular intervals and supplemented with 1 µl 0.1% Calcofluor (which reacts to cellulose in the cyst wall). Total amoeba and cyst numbers were determined by counting cells in a haemocytometer under phase contrast and UV illumination, respectively. 300–500 cells were counted for each time point.

### GSK3 kinase assay

GSK3 kinase activity was measured in cell lysates as described previously [17]. In short, *Pp* cells were resuspended at 5x10^7^ cells/ml in ice-cold lysis buffer (0.5% NP40, 10 mM NaCl, 20 mM PIPES, pH 7.0, 5 mM EDTA, 50 mM NaF, 0.1 mM Na_3_VO_4_, 0.05% 2-mercaptoethanol, 5 µg/ml benzamidine, 5 µg/ml aprotinin) and cleared by centrifugation at 10,000 × g. 5 µl cell extract was incubated for 8 min at 22 °C with 15 µl assay buffer (50 mM HEPES, pH 7.5, 4 mM MgCl_2_, 0.5 mM EGTA, 2 mM DTT, 100 µM ATP) containing 20 µg phosphoglycogen synthase peptide-2 (Upstate) and [γ-^32^P]ATP to 8–16 Bq/pmole. After the addition of 20 µl 15 mM phosphoric acid, [γ-^32^P]ATP incorporation was measured by binding to P81 phosphocellulose paper (Whatman) and scintillation counting, after extensive washing with 7.5 mM phosphoric acid. To measure non-specific phosphorylation, 50 mM LiCl (a GSK3 inhibitor) was added to the assay buffer.

## Results

### Isolation and disruption of a *GSK3* homologue in *Pp*

To identify the role of GSK3 in *Pp* development, we first amplified a full-length *GSK3* gene from *Pp* gDNA by combining PCR with degenerate primers and inverse PCR. The 2124-bp coding region contained several introns and to elucidate the gene model, we determined mRNA sequence by RT-PCR and RLM-RACE. This revealed that the *GSK3* gene consists of 5 exons and 4 introns and encodes 449 amino acids. *Pp* GSK3 shared 92% sequence identity to *D. discoideum* (*Dd*) GSK3 and 60–70% identity with GSK3s from plants, animals and other Amoebozoa (Additional file 1: Figs. 2, 3). Query of Dictyostelid genomes with *Pp* GSK3 and phylogenetic inference from alignments of the closest hits shows that *Pp GSK3* is orthologous to *Dd GSK3*.

To disrupt the *GSK3* gene in *Pp* by homologous recombination, we transformed *Pp* with a construct in which the floxed A15neoR cassette is flanked by two fragments of the *GSK3* gene, and obtained two *gsk3* null clones from about 1000 G418-resistant clones (Additional file 1: Fig. 1). To confirm that the phenotype of the *gsk3*^−^ mutant was due to the loss of *GSK3*, the G418 resistance cassette of the mutant was removed by transformation with Cre-recombinase and a complementation vector, which contains *Pp GSK3* inclusive of its promoter, was introduced into the disruptant.

### Growth phenotype of the *Pp gsk3* null mutant

**Fig. 1:**
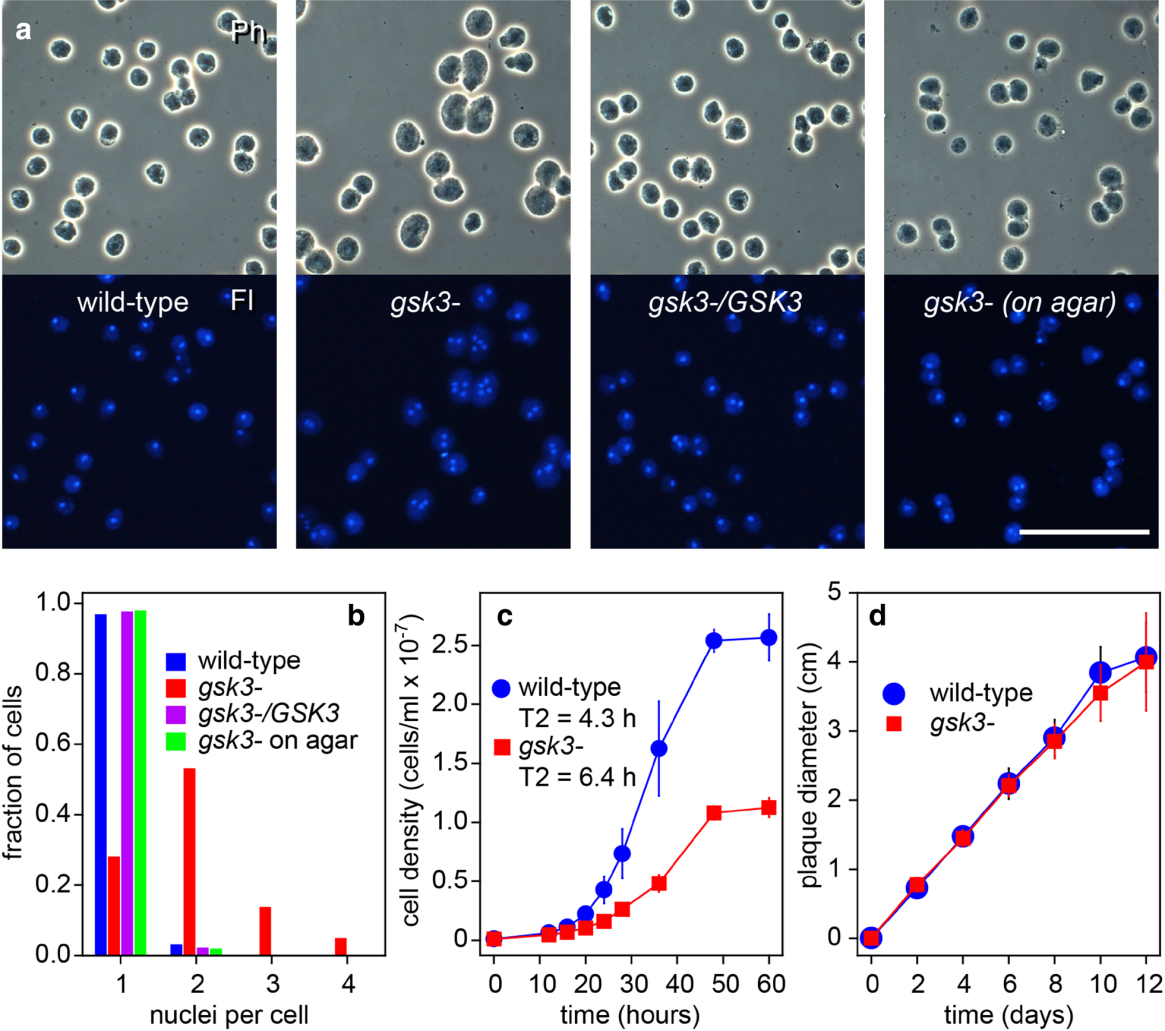
Effect of deletion of *Pp GSK3* on cell division. **a** *Cell division. P. pallidum* wild-type, *gsk3*^−^ and *gsk3*^−^/*GSK3* cells were inoculated at 10^5^ cells/ml in KK2 with autoclaved *K. aerogenes* and shaken at 22 °C until amoebas had reached late log phase. Cells were then stained with 0.01% DAPI and photographed under phase contrast (Ph, upper panels) and epifluorescence (Fl, lower panels). *gsk3*^−^ cells were also grown on agar plates with live bacteria and then stained with DAPI (right panels). Bar: 50 µM. **b** *Nuclei per cell.* For about 150 cells per strain, the number of nuclei per cell was counted by comparing phase contrast and DAPI fluorescence images, and the fraction of cells with 1, 2, 3 or 4 nuclei was calculated. **c** *Growth in suspension.* Wild-type and *gsk3*^−^ cells were grown in suspension on autoclaved *K. aerogenes* as described above. At the indicated time points, the cell density was determined and doubling times (T2) were calculated from the exponential phase of the growth curve. Means and SD of 4 experiments are presented. **d** *Growth on agar.* Wild-type and *gsk3*^−^ spores were clonally plated on nutrient agar with live bacteria, and the diameter of emerging plaques in the bacterial lawn was measured with a ruler at 2-day intervals. Means and SD of 4 experiments

The *D. discoideum* (*Dd*) *gsk3*^−^ mutant becomes multinucleate due to a cytokinesis defect, when grown in suspension in axenic medium, but not when grown on agar with bacteria as food source [20]. *Pp* grows poorly in axenic medium, but can be grown in suspension on autoclaved bacteria. We first tested whether *Pp gsk3*^−^ shows a similar cytokinesis defect as *Dd gsk3*^−^. When grown in suspension with autoclaved *Klebsiella aerogenes*, the *Pp gsk3*^−^ cells seemed larger than wild-type cells. DAPI staining revealed that most *gsk3*^−^ cells contained multiple nuclei, while most wild-type cells contained a single nucleus (Fig. 1a). Complementation of *gsk3*^−^ with *GSK3* restored the mononucleate phenotype. When the *Pp gsk3*^−^ mutant was grown with bacteria on agar, cells remained mononucleate and had the same size as wild-type cells (Fig. 1a). Counting of the number of nuclei per cell showed that less than 5% of wild-type, *gsk3*^−^/*GSK3* or *gsk3*^−^ cells grown on agar had more than one nucleus per cells, while 72% of *gsk3*^−^ cells grown in suspension had two or more nucleo (Fig. 1b). The cytokinesis defect also reduced the doubling time of *gsk3*^−^ cells grown in suspension from 6.4 h to 4.3 h as well as the cell density reached at stationary phase (Fig. 1c). However, proliferation of *gsk3*^−^ cells on solid substratum was normal, as judged from the increase in plaque size of *gsk3*^−^ cells grown clonally on agar with bacterial lawns (Fig. 1d). Evidently, like *Dd gsk3*^−^, *Pp gsk3*^−^ shows defective cytokinesis when grown in suspension, but not on solid substratum, indicating that the requirement of GSK3 for proper cytokinesis is conserved in Dictyostelia.

### Developmental phenotype of the *Pp gsk3* null mutant

**Fig. 2:**
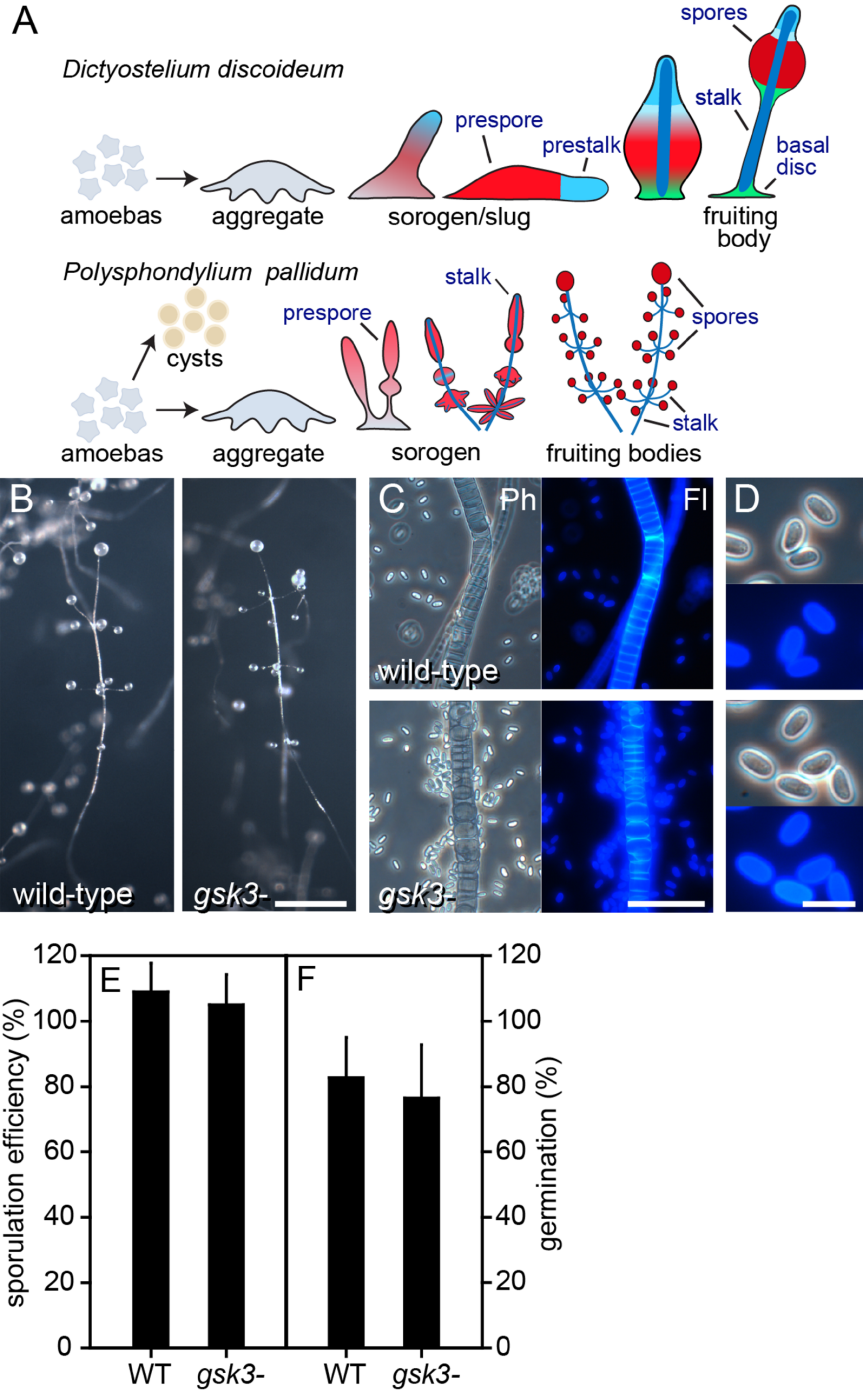
Developmental phenotype of the *Pp gsk3*^−^ mutant. **A** *Cartoon highlighting differences between Pp and Dd development.* **B** *Fruiting bodies. Pp* wild-type and *gsk3*^−^ cells were freed from bacteria and developed for 24 h on non-nutrient agar at 10^6^ cells/cm^2^ and photographed. Bar: 0.5 mm. **C** *Stalk cells*. The fruiting bodies were picked up, deposited in 0.001% Calcofluor on a slide glass and photographed under phase contrast (Ph) and epifluorescence (Fl) Bar: 50 µm. **D** *Spores*. Prepared as in panel B, but photographed at higher magnification. Bar: 10 µm. **E** *Sporulation efficiency.* 4 × 10^6^ freshly harvested *Pp* amoebas were plated on 2 × 2 cm^2^ nitrocellulose membranes, supported by non-nutrient agar. After completion of fruiting body formation, membranes were vortexed in 0.1% Triton-X100 and spore numbers were counted and expressed as percentage of plated cell numbers. Means and SD from 3 independent experiments. **F** *Spore viability*. *Pp* spores were harvested from 7-day-old fruiting bodies, treated for 10 min with 0.1% Triton-X100 to remove amoebas, counted and clonally plated on LP agar with *E. coli.* Emerging *Pp* colonies were counted after 4–5 days. Means and SD from 4 independent experiments. There were no significant differences between wild-type (WT) and *gsk3*^−^ cells in panels D and E (*t* test, *P* > 0.5)

*Pp* development differs from that of *Dd* in several aspects (Fig. 2A). There is no migrating slug stage with prestalk cells as in *Dd*. *Pp* cells first all differentiate into prespore cells, only to dedifferentiate into stalk cells at the tips of primary and secondary sorogens. There are also no basal disc cells to support the stalk. However, while *Dd* fruiting bodies are unbranched, *Pp* forms regular whorls of side branches out of cell masses that pinch off from the rear of the primary sorogen. Additionally, *Pp* has retained the ancestral survival strategy of encystation of individual amoebas under conditions that are unfavourable for aggregation, which is lost in *Dd*.

The *Dd gsk3*^−^ mutant forms abnormal fruiting bodies with a large basal mass of stalk-like cells and relatively few spores [16]. However, *Pp gsk3*^−^ cells appeared to form normal fruiting bodies with a neat array of stalk cells and the multiple spore heads that are common to this species (Fig. 2B). Both the spore and stalk walls stained positively with Calcofluor White, a compound that fluoresces when interacting with cellulose, indicating that they had properly reached their mature cellulose-encapsulated state (Fig. 2C). The elliptical spores of the *Pp gsk3*^−^ mutant were morphologically indistinguishable from those of wild-type cells (Fig. 2D). We determined sporulation efficiency of the *gks3*^−^ mutant by counting the number of spores differentiating from a known number of amoebas. Figure 2E shows that the *Pp gsk3*^−^ cells sporulated as efficiently as wild-type cells. Spore numbers for both exceeded that of plated amoebas, which is due to some cell division still occurring after plating the cells. Spore viability was also normal in *Pp gsk3*^−^, since *Pp gsk3*^−^ spores germinated as efficiently as wild-type spores (Fig. 2F).

**Fig. 3:**
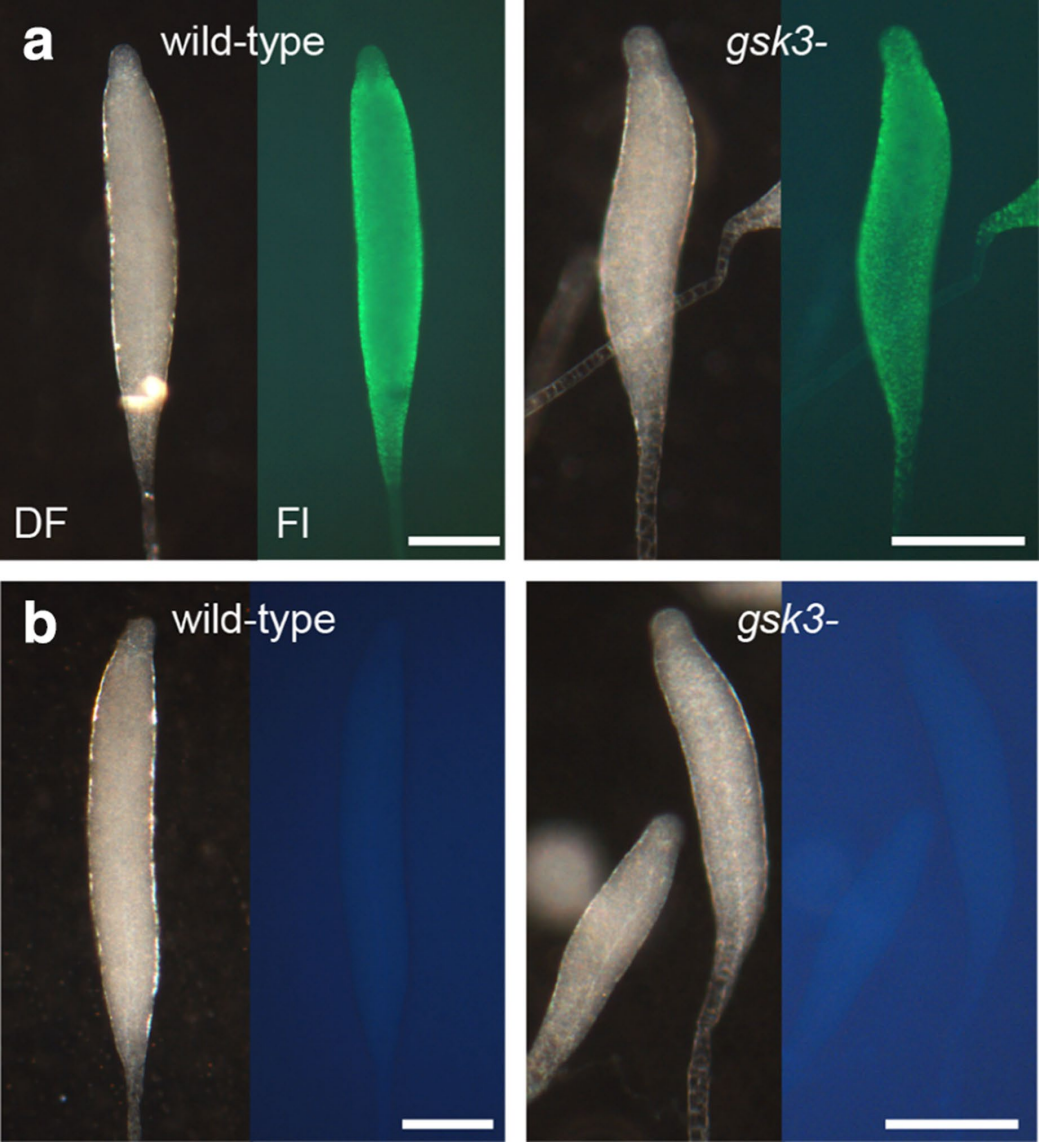
Prespore differentiation in sorogens. *Pp* wild-type and *gsk3*^−^ sorogens were fixed and stained with rabbit antispore antibodies and FITC-conjugated antirabbit-IgG [39] (**a**) or only with FITC-conjugated antirabbit-IgG (**b**). Structures were photographed under dark field (DF) and epifluorescence (Fl), with prolonged exposure for the structures in **b**. Bar: 0.1 mm

Using antispore serum, which apart from spores also detects vesicles with prefabricated spore coat components in prespore cells [20], we assessed whether *Pp gsk3*^−^ cells normally differentiate in prespore cells. Figure 3 shows that both wild-type and *gsk3*^−^ cells show the same pattern of spore antigen expression, with only the utmost tip of the sorogen and the stalk devoid of spore antigen as is the norm for this species [4] (Fig. 2a). These experiments indicate that GSK3 is not required for either prespore or spore differentiation in *Pp*.

### Encystation of *gsk3* null mutant

**Fig. 4:**
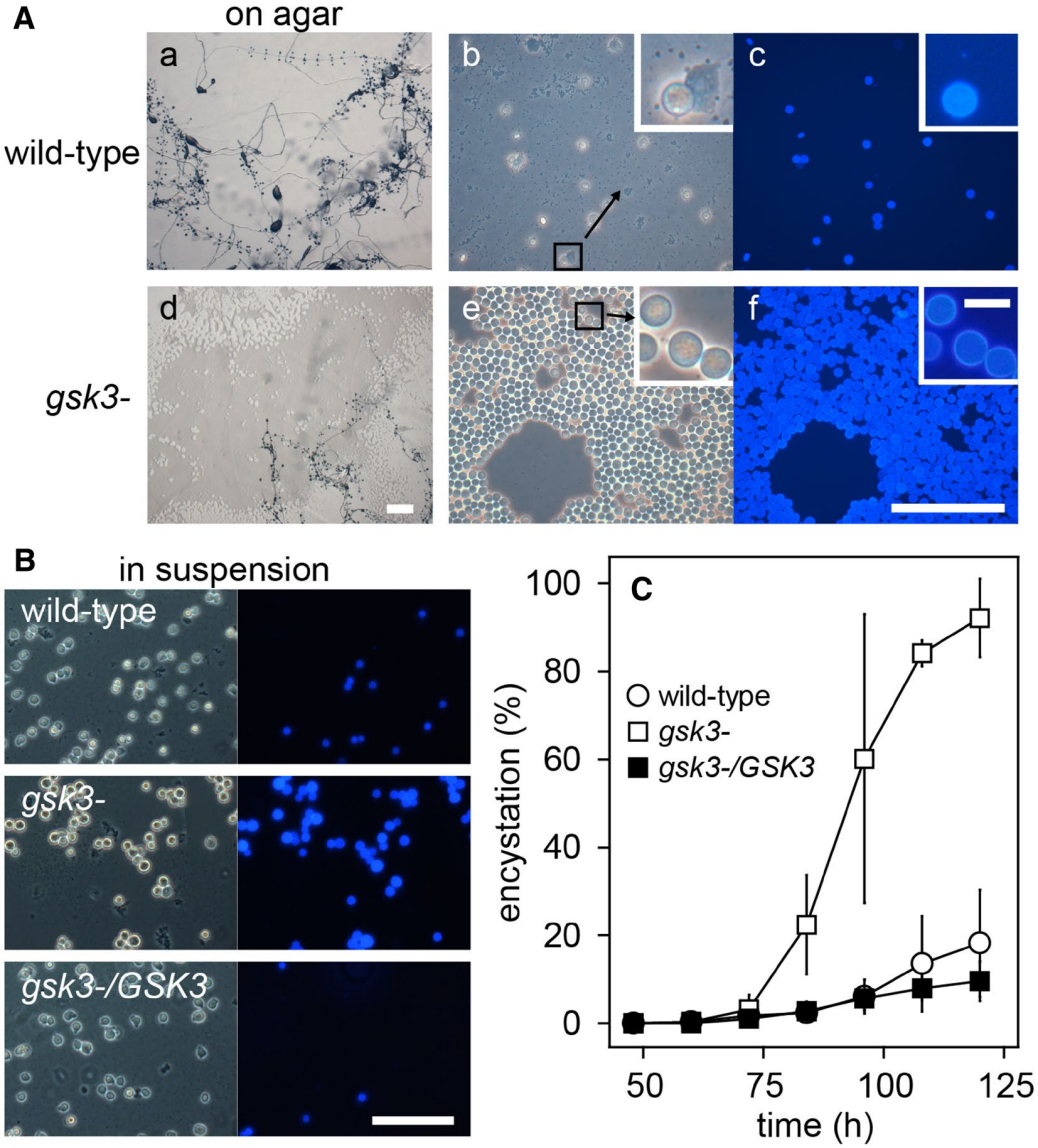
Development on nutrient agar and in suspension. **A** *On agar.* Wild-type and *gsk3*^−^ cells were cultured in darkness with live *E. coli* on nutrient agar until, about 24 h after clearing bacteria, fruiting bodies had fully formed. Plates were photographed at low magnification (left, Bar: 1 mm). The agar surfaces were then wetted with 0.001% Calcofluor and photographed under phase contrast (centre) and epifluorescence (right). Bar: 100 µm, inset bar: 10 µm. **B** *In suspension.* Wild-type, *gsk3*^−^ and *gsk3*^−^/*GSK3* cells were incubated in suspension with autoclaved *K. aerogenes* for 120 h, with bacteria being cleared at 50–60 h. Cells were then stained with Calcofluor and photographed under phase contrast and UV illumination. Bar: 100 µm. **C** *Quantitation.* From the experiment shown in **B**, cells were sampled at the indicated time points and stained with Calcofluor. The numbers of fluorescent cysts and unstained amoebas were counted under UV and phase contrast illumination, respectively, and the percentage of cysts over total cells was calculated. Means and SD of 3 independent experiments, each counting 300–500 cells/sample, are presented

When, as in the experiments above, *gsk3*^−^ cells are freed from bacteria and then plated on non-nutrient agar, the greater majority of amoebas aggregate and become incorporated into fruiting bodies with similar timing as wild-type cells. However, when cells are left on the culture plate after the bacteria have been eaten, we observed that many *gsk3*^−^ cells did not aggregate and develop into fruiting bodies, but remained on the agar surface. This occurred particularly when plates were kept in the dark, where wild-type cells still developed normally, leaving very few cells behind on the agar surface (Fig. 4Aa–c). The prostrate *gsk3*^−^ cells (the large opaque area in Fig. 4Ad) were round and refractile (Fig. 4Ae), and Calcofluor White staining (Fig. 4Af) revealed that they had cellulose walls and were actually microcysts.

We also observed that *gsk3*^−^ cells formed microcysts more readily after consumption of autoclaved *K. aerogenes* when grown in suspension (Fig. 4B). Both wild-type and *gsk3*^−^ cells reach stationary phase after about 48 h in suspension culture (Fig. 1c) with all bacteria being consumed within 60 h. Both wild-type and *gsk3*^−^ cells started to encyst around 60–72 h (Fig. 4C). After 120 h only 20% of wild-type cells had encysted as opposed to 90% of *gsk3*^−^. Complementation of *gsk3*^−^ cells with *GSK3* reduced their ability to encyst to that of wild type, indicating that loss of *GSK3* potentiates encystation.

### Production of and response to cyst-inducing factors

**Fig. 5:**
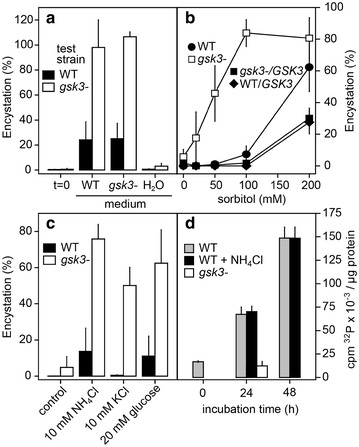
Effects of secreted factors and osmolytes on encystation and GSK3 activity. **a** *Secreted factors.* Wild-type and *gsk3*^−^ cells were grown to stationary phase with autoclaved *K.aerogenes*, and medium and cells were separated by centrifugation. The cells were subsequently incubated for 60 h in either water, wild-type medium or *gsk3*^−^ medium, and cyst percentages were determined after staining with Calcofluor. Means and SE of 4 experiments are presented. **b** *Sorbitol.* Wild-type, *gsk3*^−^, *gsk3*^−^/*GSK3* and wild-type/*GSK3* cells were incubated with the indicated concentrations of the osmolyte sorbitol. After 48 h of incubation, cells were stained with Calcofluor and percentages of fluorescent cysts were determined. Means and SE of 3 experiments. **c** *Osmolyte sensitivity.* Wild-type and *gsk3*^−^ cells were incubated in 10 mM NH_4_Cl, 10 mM KCl or 20 mM glucose for 48 h. After Calcofluor staining, the percentage of fluorescent cysts was determined. Means and SE of 4 experiments. **d** *GSK3 activity.* Wild-type cells were incubated with and without 10 mM NH_4_Cl and *gsk3*^−^ cells without NH_4_Cl only. Cell extracts were prepared at the indicated time points and incubated with [γ-^32^P]ATP and the GSK3 substrate phosphoglycogen synthase peptide-2 in the presence and absence of the GSK3 inhibitor LiCl. After 8 min, ^32^P incorporation in the peptide was measured and non-specific ^32^P incorporation in the presence of LiCl was subtracted [17]. Means and SE of 4 experiments are presented

The increased ability of *gsk3*^−^ cells to encyst could either be due to *gsk3*^−^ cells producing more of an encystation-inducing factor or to being more sensitive to such a factor. To test the possibility that *gsk3*^−^ cells produce more of an encystation-inducing factor, we prepared supernatants from suspension cultures of either wild-type or *gsk3*^−^ cells at 60 h of culture, when bacterial food is just depleted. Supernatants prepared from both wild-type and *gsk3*^−^ cultures strongly induced encystation of *gsk3*^−^ cells, but were much less effective in wild-type cells. Incubation with water as control was ineffective to induce encystation of *gsk3*^−^ cells (Fig. 5a). Also phosphate buffer and medium prepared from heat-killed *K. aerogenes* were ineffective to induce encystation (data not shown). These results indicate that *gsk3*^−^ cells responded more strongly to encystation-inducing factor(s), rather than secreting more of such factors and that their encystation tendency is cell autonomous.

Osmolytes were previously reported to effectively induce encystation [21], with NH_4_Cl and KCl being more effective than other ions or solutes [22]. We compared the effects of a range of osmolytes on encystation in *gsk3*^−^ and wild-type cells. In the presence of 200 mM sorbitol, about 60% of wild-type cells encysted after 48 h of incubation, against 80% of *gsk3*^−^ cells (Fig. 5b). At 100 mM sorbitol, only 10% of wild-type cells encysted, but still 80% of *gsk3*^−^ cells, while 50 mM did not induce wild-type encystation anymore, but still caused 50% of *gsk3*^−^ cells to encyst (Fig. 5b). On the other hand, *gsk3*^−^ cells complemented with *GSK3* and wild-type cells overexpressing *GSK3* were even less responsive to sorbitol than wild-type cells. Similar results were obtained with other osmolytes. Both 10 mM of KCl and NH_4_Cl and 20 mM glucose induced 50–80% encystation of *gsk3*^−^ cells, compared to 1–13% of wild-type cells (Fig. 5c). These findings indicate that *gsk3*^−^ cells were much more sensitive to osmolytes than wild-type cells. While the naturally secreted encystation-inducing factor is unknown, NH_3_/NH_4_^+^ is a candidate, because NH_3_ is produced in large amounts by protein degradation in starving cells.

We next measured whether NH_4_Cl affects GSK3 activity, measured in cleared lysates of wild-type cells starved in suspension buffer. Figure 5d shows that the ability of GSK3 to phosphorylate phosphoglycogen synthase peptide-2 increases up to fourfold after 24 h of starvation and up to eightfold after 48 h. However, 10 mM NH_4_Cl had no effect on GSK3 activity. The *gsk3*^−^ cells showed only 10.8 ± 1.8% of the kinase activity of wild-type cells after 24 h of starvation, indicating that non-specific phosphorylation of phosphoglycogen synthase peptide-2 in this experiment was low. This experiment shows that GSK3 is not itself regulated by NH_4_Cl, but may inhibit the pathway that mediates NH_4_Cl-induced encystation.

## Discussion

### GSK3 has a conserved role in cytokinesis of Dictyostelia

*Pp* *GSK3* null mutant cells were larger than those of wild type and contained multiple nuclei, indicative of a defect in cytokinesis (Fig. 1). When grown on solid substratum, these defects were not observed. This behaviour is also found in the *Dd gsk3*^−^ mutant [23] and in several *Dd* mutants in cytoskeletal proteins [24–26]. In both *Dd* and animals, GSK3 associates with the mitotic spindle during cell division [23, 27] and has for animals been described to phosphorylate microtubule-associated proteins, with the loss of GSK3 activity causing defects in spindle alignment [27, 28]. Apparently, this role of GSK3 is deeply conserved between animals and Dictyostelia. On solid substratum, *Dictyostelium* cells can also undergo cytokinesis by actin-based traction forces [26], a process which likely does not require GSK3.

### Loss of GSK3 does not affect prespore and spore differentiation in *Pp*

Unlike the *Dd gsk3*^−^ mutant in strain DH1 [16], the *Pp gsk3*^−^ mutant showed no defects in prespore or spore differentiation (Figs. 2, 3). In *Dd*, the cAMP receptor cAR3 and the tyrosine kinases Zak1 and Zak2 are considered to mediate cAMP induction of prespore gene expression by GSK3 by phosphorylating GSK3 at tyrosine residues Y214 and Y220. Conversely, cAMP acting on cAR4 suppresses GSK3 activity by stimulating a tyrosine phosphatase [17, 29–31]. The *Dd* DH1/*gsk3*^−^ fruiting bodies consist mostly of a basal mass of stalk-like vacuolated cells and few spores, but this extreme phenotype was not observed in a *GSK3* knockout in *Dd* strain AX2, which forms fruiting bodies with shorter stalks, but normal spores [32]. cAMP-induced prespore gene expression is normal in AX2/*gsk3*^−^, but the mutant is hypersensitive to induction of the stalk marker gene *ecmB* by DIF-1, and like the DH1/*gsk3*^−^, *car3*^−^ and *zak1*^−^ mutants, does not show cAMP inhibition of *ecmB* [17, 29, 30, 32]. This suggests that GSK3 indirectly favours the spore pathway in *Dd* by preventing its inhibition by DIF-1. DIF-1 was originally identified as the stalk-inducing factor of *Dd*, but deletion of its biosynthetic pathway revealed that it was not required for the differentiation of the stalk, but of the basal disc [33–35]. The basal disc cells are phenotypically identical to stalk cells and also express *ecmB* [36]. Species outside of group 4, including *Pp*, do not form a basal disc and have neither *cAR3* nor *Zak1* or *Zak2* in their genomes [10, 37]. It therefore appears that the role of GSK3 in regulating prespore/basal disc proportions newly evolved in group 4.

### GSK3 controls the decision between encystation and fructification in *Pp*

Earlier studies showed that similar to *Dd* [5], PKA is required for entry into multicellular development and for spore and stalk maturation in *Pp*, and additionally for entry into encystation [7, 11], suggesting that PKA’s roles in multicellular development are evolutionary derived from a more ancestral role in encystation. On the other hand, GSK3 appears to control the decision to either encyst or aggregate and form fruiting bodies. The *Pp gsk3*^−^ mutant formed cysts under conditions where wild-type cells normally aggregate and was hypersensitive to secreted factors and osmolytes that induce encystation (Figs. 4, 5). However, GSK3 activity was itself not regulated by these factors. GSK3 was also not obviously regulated at the expression level, since it is already present in feeding amoebas and modestly upregulated during both encystation and multicellular development (Additional file 1: Fig. 2). In *Dd,* GSK3 is required for the upregulation of about 81 genes and downregulation of 105 others in early development [38]. The hypersensitivity of *Pp gsk3*^−^ cells to encystation-inducing factors might occur if sensors for these factors or components of their signal transduction pathways were among the *Pp* genes downregulated by GSK3.

## Conclusions

Most Dictyostelia can choose between solitary encystment and social sporulation in fruiting bodies when faced with environmental stress. We show that active GSK3 favours sociality over solitary survival.

In contrast to *D. discoideum* where GSK3 promotes prespore differentiation by inhibiting basal disc differentiation, GSK3 has no effects on prespore or spore differentiation in the encysting Dictyostelid *P. pallidum*, most likely because they do not form the basal disc.

It is possible that the lack of encystment in *D. discoideum* and group 4 in general, allowed recruitment of the GSK3 pathway for regulating the differentiation of a novel encapsulated cell type in the group, i.e. the basal disc.

## Additional file

**Additional file 1. ** Additional figures 1–3 and additional table 1.
