# Supplementary material for: Glycogen synthase kinase 3 promotes multicellular development over unicellular encystation in encysting Dictyostelia
Source: EvoDevo. 2018 May 9;9:12. doi: 10.1186/s13227-018-0101-6 (PMC5941370; doi:10.1186/s13227-018-0101-6)
Supplement: Supplementary file 1 — Additional file 1. Additional figures 1–3 and additional table 1. [file 13227_2018_101_MOESM1_ESM.docx]

**Additional File (Figures 1-3, Table 1)**

**
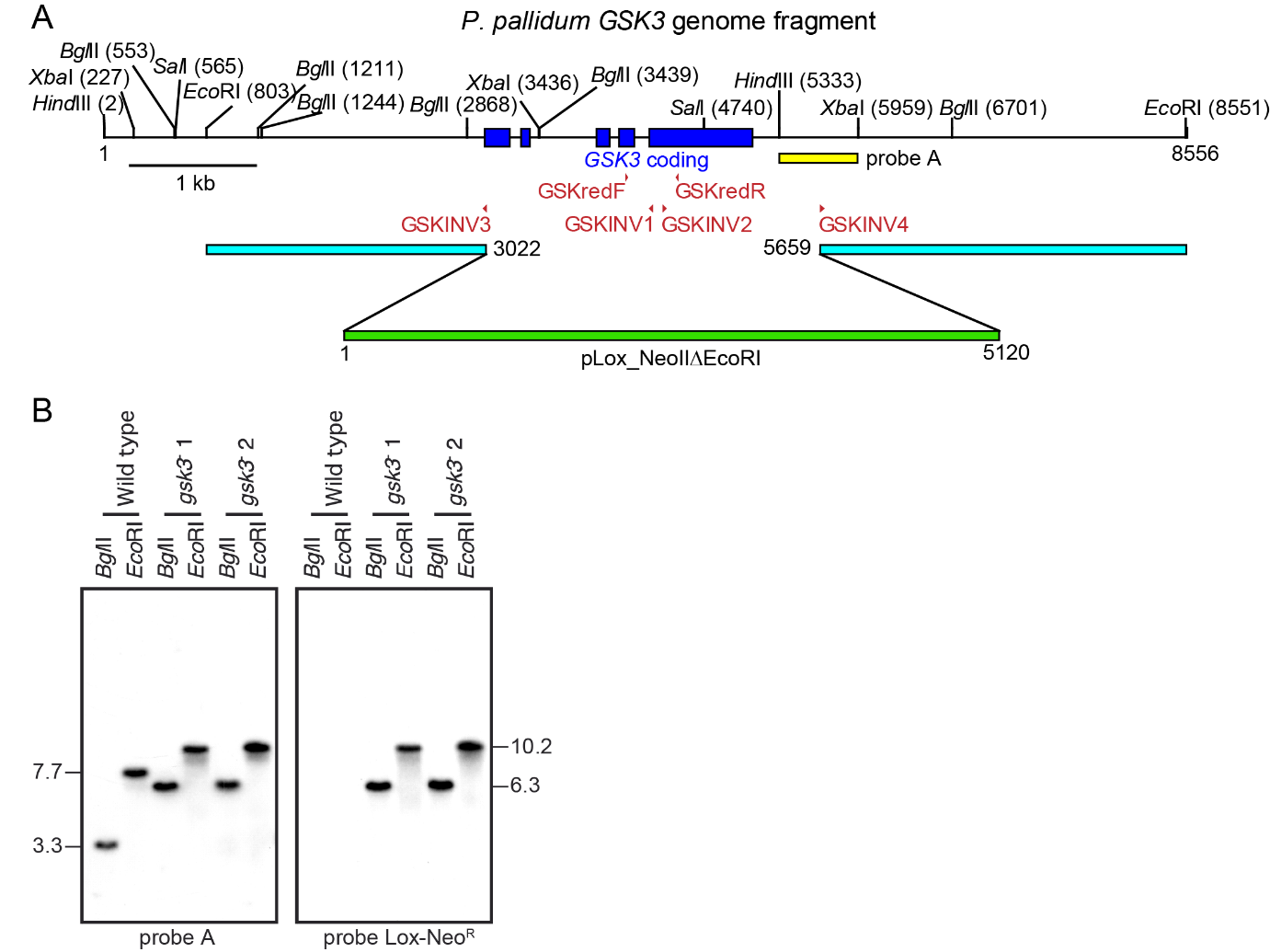
**

**Additional Figure 1. Cloning and knock-out of *Pp GSK3***

A. *Pp GSK3 cloning and knock-out construct.* A fragment of the GSK3 gene was amplified by PCR with degenerate primers GSKredF and GSKredR, and partially overlapping 5.3 kb and 3.3 kb genomic fragments were subsequently amplified by inverse PCR using primers GSKINV1 and GSKINV2 of *Hind*III or *Bgl*II digested and re-ligated genomic DNA, respectively. A knock-out construct was prepared by inverse PCR of *Eco*RI digested and re-ligated gDNA using primers GSKINV3 and GSKINV4 and cloned into the *Kpn*I site of pLoxNeoIIΔ*Eco*RI, which after *Eco*RI digestion was transformed into *Pp* cells.

B. *GSK3 knockout diagnosis.* Genomic DNAs from G418 resistant clones and wild-type cells were digested with *Eco*RI or *Bg*lII and Southern blots were probed with DNA probe A complementary to the GSK3 gDNA (left panel), showing the expected 2.5 and 3.0 kb size increase of *Eco*RI and *Bgl*II fragments compared to wild-type due to homologous recombination. Hybridization of the Southern blot to a probe complementary to the NeoR gene shows the absence of NeoR in wild-type cells and its presence in the knock-outs (right panel).

**
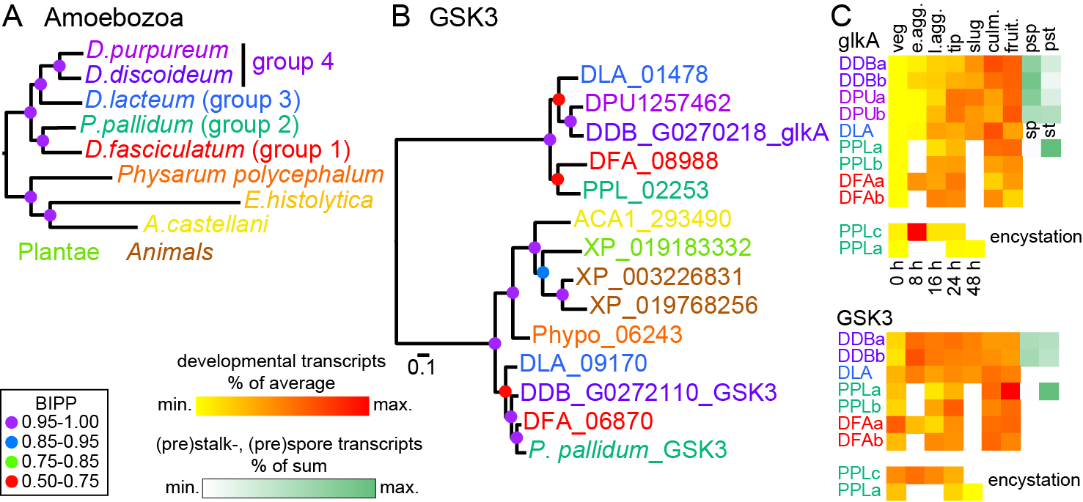
**

**Additional Figure 2. Dictyostelid homologs of GSK3 and their developmental regulation**

A. Phylogenetic tree of Dictyostelia inferred from 32 concatenated proteins retrieved from genomes representative of the four major groups of Dictyostelia and three genomes of unicellular Amoebozoa [1]. Bayesian posterior probabilites of the nodes are indicated by coloured dots.

B. Phylogenetic tree inferred by Bayesian inference from the closest homologs of *P.pallidum* GSK3 in Dictyostelia, unicellular Amoebozoa, animals and plants. The Dictyostelia each additionally contain a GSK3-like (*glkA*) gene, but BLASTp searches with this gene in animals yielded XP_019768256 (GSK3_β, *Dendroctonus ponderosae) and* XP_003226831 (GSK3_β, *Anolis carolinensis*) as top hits, which group with the “real” GSK3 orthologs.

C. Developmental expression profiles and cell-type specifity of the two sets of Dictyostelid genes were retrieved from published RNA sequencing experiments [2, 3]. The data show that GSK3 is already present in vegetative cells and shows a modest increase during development to fruiting bodies and encystation, while the GSK3-like genes show a more pronounced increase in early development.

**HUMAN_GSK3B**  **-----------------------------------MHHHHHHHHHHKVSRDKDGSKV-------------** 22

**Ddisco_GSK3**  **-----------------------------------MSSKDQILEKDKKETDDNGNKKTTTTTSSSSSSSS** 35

**PpalGSK3**  **------------------------------------------------------------MSTKEDEKEK** 10

**DDB_G0270218** **MTIPTDNNSSNNKGYNDNNNNNNNNNNNNNNNNNNNNNNNKEHPNINNNNNNNNNNNNNNIKESSSSNSN** 70

**HUMAN_GSK3B**  **--TTVVATPGQGPDRPQEVSYTDTKVIGNGSFGVVYQAKLCDSGELVAIKKVLQDKRFKNRELQIMRKLD** 90

**Ddisco_GSK3**  **SSKPRSNKFDKVIIKSNGVCYITEGVIGNGSFGVVTQAIVADTKEVVAIKKVLQDQRYKNRELQIMKMLN** 105

**PpalGSK3**  **NKTKLRNKFDKVIIKSNGVCYITEGIIGNGSFGVVTQAIVSDTKEVVAIKKVLQDHRYKNRELQIMKMLN** 80

**DDB_G0270218** **HSSSQSSSTATVNSNPKVYPYEIIKQVGQGTFGKVYEAKNQDNK-RVAIKKVEKSNHFISREYDILKIVA** 139

**HUMAN_GSK3B**  **HCNIVRLRYFFYSSGEKKDEVYLNLVLDYVPETVYRVARHYSRAKQTLPVIYVKLYMYQLFRSLAYIHSF** 160

**Ddisco_GSK3**  **HINIVSLKNSFYTSD--NDEVYLNLVLEYVPDTVYRVSRHYSMSKQPVPNIFVKLYIYQLCRSINYIHSL** 173

**PpalGSK3**  **HINIVQLKNSFMTSD--NDEVYLNLVLEFVPDTVYRVSRHYTTSKQSIPLIYVKLYIYQLCRAISYIHSL** 148

**DDB_G0270218** **HPNCLRILDMFYTAE--DNKKMQNLVFDFIPYTLASLL-----KKRQLSINFIKVLFYQLCQAIKHIHSK** 202

**HUMAN_GSK3B**  **GICHRDIKPQNLLLDPDTAVLKLCDFGSAKQLVRGEPNVSYICSRYYRAPELIFGATDYTSSIDVWSAGC** 230

**Ddisco_GSK3**  **GICHRDIKPQNLLLDTSTSTLKLCDFGSAKILIKGETNVSYICSRHYRAPELIFGSTNYTTTIDVWSLGC** 243

**PpalGSK3**  **GICHRDIKPQNLLLDSQTSILKLCDFGSAKILIKDEANVSYICSRHYRAPELIFGSTNYTTSIDVWSLGC** 218

**DDB_G0270218** **AICHRDITPNNILLSS-KGELTLADFGSAKILESNHTSMSYICSRYYRAPELLVGCSNYTTKIDIWSIGC** 271

**HUMAN_GSK3B**  **VLAELLLGQPIFPGDSGVDQLVEIIKVLGTPTREQIREMNPNYT-EFKFPQIKAHPWTKVFRPRT-PPEA** 298

**Ddisco_GSK3**  **VLAELLLGQPLFPGENGIDQLVEIIKVLGTPTKEQIHAMNPYYT-SFKFPEIKANPWPRVFKAKDVPAES** 312

**PpalGSK3**  **VLAELLLGAPLFPGENGIDQLVEIIKVLGTPTKEQIHVMNPYYS-SFKFPDIKANPWTKVFRAKDVPAEA** 287

**DDB_G0270218** **ILAEMLIGKPLFPGTNSNDQLGRIIEVLGSPTKDDMEAMKPSKPYHLQLPNINPKFFESLHNVE--DKTV** 339

**HUMAN_GSK3B**  **IALCSRLLEYTPTARLTPLEACAHSFFDELRDPNVKLPNGRDTPALFNFTTQE-LSSNPPLATILIPPHA** 367

**Ddisco_GSK3**  **IDLISKILLYDPSSRLKPVEICAHPFFDELRDPKTCLPDGKPLPPLFNFTIAEQTSIGPKLAKTLIPSHA** 382

**PpalGSK3**  **IDLISKILHYDPSARLKPTEICAHPFFDELRDPKSTLPDGRPLPPLFNFTIAEQLTMGPKLAKILIPPHA** 357

**DDB_G0270218** **VDLLSKIFIFDPVKRASIDEIIAHPFLRDVNINSLELFDEMK-----CFSVSGNGKSSLTTN--------** 396

**HUMAN_GSK3B**  **RIQAAASTPTN-----------------------------------------------------------** 378

**Ddisco_GSK3**  **MNQIELPSPLFPNLAISSSNQSSSSN------------SNANVSSNLNSHSASPSTTSSSSSTPNSIPVQ** 440

**PpalGSK3**  **MSAIELPSPLFPHLTSGAPVSSSSTT------------TPTPSPSNHST------SNSSITSSTTNIQAQ** 409

**DDB_G0270218** **---STSSSSTTANMTSLASSSSNNKTTCSETYLSRLPTSAITSSSNLKSIDN--SNNGKSSSSSNNIPSL** 461

**HUMAN_GSK3B**  **----------------------------------------** 378

**Ddisco_GSK3**  **SPSTTNTTSSTTNNTTTTTTTTTTSNH-------------** 467

**PpalGSK3**  **SSSSSTSQTTSNSTNTQTTAQANPTTTTTTNTVGTSTNAN** 449

**DDB_G0270218** **NNSNNGVITNTI----------------------------** 473

**Additional Figure 3. GSK3 alignment**

Alignment of human GSK3β, *D. discoideum* and *P. pallidum* GSK3 and the *D.discoideum* GSK3-like gene DDB_G0270218. Grey shading: identical residues; yellow shading: binding to axin of human GSK3; green shading: polypeptide substrate binding site; red shading: phosphorylated by ZAK1 and essential for GSK3 activity [4].

**Additonal Table 1. Oligonucleotide primers used in this work**

| **Name** | **DNA sequence** |
| --- | --- |
| GSKredF | TGYCAYAGRGAYATHAARCC |
| GSKredR | GYTCYYTIGTIGGNGTNCC |
| GSKINV1 | CATTCGCTTCGTCTTTAATAAGAATCTTGGCAC |
| GSKINV2 | ATGTTTGGTCGTTGGGATGTGTTTTGGCAGA |
| GSKINV3 | ggggtacCGTCTTCCTTTGTACTCATTTTTGGTGGTG |
| GSKINV4 | ggggtaccgagCTCTCTCTCCACTCTATACATTCACCAGATCAAC |
| Pp-GSK3-S51 | AGATCTTTAAGTCATTTTGTCAGCATTACAC |
| Pp-GSK3-E31E | ggaattcGTTTGCATTTGTACTTGTTCCAACTGTG |
| Pp-GSK3-51 | GATAGTCACAATCATGCT |
| Pp-GSK3-31 | GAATGGTGAATATGGACT |

**ADDITONAL REFERENCES**

1. Romeralo M, Skiba A, Gonzalez-Voyer A, Schilde C, Lawal H, Kedziora S, Cavender JC, Glockner G, Urushihara H, Schaap P: Analysis of phenotypic evolution in Dictyostelia highlights developmental plasticity as a likely consequence of colonial multicellularity. Proc Biol Sci 2013, 280(1764):20130976.

2. Parikh A, Miranda ER, Katoh-Kurasawa M, Fuller D, Rot G, Zagar L, Curk T, Sucgang R, Chen R, Zupan B *et al*: Conserved developmental transcriptomes in evolutionarily divergent species. Genome Biol 2010, 11(3):R35.

3. Glockner G, Lawal HM, Felder M, Singh R, Singer G, Weijer CJ, Schaap P: The multicellularity genes of dictyostelid social amoebas. Nature communications 2016, 7:12085.

4. Kim L, Harwood A, Kimmel AR: Receptor-dependent and tyrosine phosphatase-mediated inhibition of GSK3 regulates cell fate choice. Dev Cell 2002, 3(4):523-532.
